# Retrospective Study of Platelet-Rich Plasma and Its Uses in Knee Pain in a District General Hospital in the United Kingdom

**DOI:** 10.7759/cureus.97126

**Published:** 2025-11-17

**Authors:** Surya Prasad, Adnan Faraj

**Affiliations:** 1 Orthopaedic Surgery, York Hospital, York, GBR; 2 Orthopaedics, Scarborough General Hospital, Scarborough, GBR

**Keywords:** cost saving, intra-articular steroids, knee injection, orthopaedic department, osteoarthritis (oa), persistent knee pain, platelet-rich plasma/prp

## Abstract

Introduction

Knee pain and knee osteoarthritis (kOA) are increasingly prevalent pathologies presenting to primary care and secondary care alike, contributing to worsening financial burdens shouldered by the NHS. Platelet-rich plasma (PRP) is an established option in the management of knee pain. This study primarily aims to analyse the use of intra-articular PRP injections in the treatment of knee pain, especially kOA.

Material and methods

A total of 43 patients who visited the elective clinics between 1 January 2024 and 1 January 2023 over a 12-month period and underwent PRP injection treatment for knee pain were identified, and data were obtained through the computerised patient data. All patients included in the study had already trialled simple analgesics and conservative measures with no success. PRP therapy was used in conjunction with the analgesics and conservative measures. Pain relief duration following the injection was studied. Analysis included whether these patients could be successfully discharged, defined as discharged without further recall or attendance, only needed a telephone follow-up (TFU), or had to be listed for further treatment (including arthroscopy, repeated intra-articular injections, imaging, or arthroplasty). The patients who were discharged were able to manage their pain after the PRP successfully with simple analgesia, whereas prior to the PRP, they were unable to.

Results

The number of women presenting at the clinic was significantly higher than men (33 women vs 10 men, p < .00001). A total of 37 out of 43 patients had the full three-course of injections (86.05%), three patients had two injections (6.98%), and three patients only needed one injection (6.98%). After the respective course of injections, 14 patients (32.56%) were successfully discharged from the clinic, and a further six only needed TFU (13.95%). A total of 16 patients (37.21%) needed a further face-to-face follow-up (FTF) or investigations, and three patients (6.98%) needed further PRP injections. Four patients (9.30%) were subsequently listed for a total knee replacement (TKR) despite the PRP injections.

Conclusion

PRP remains a valid option in the management of knee pain; the efficacy and the duration of pain relief, however, are unpredictable. Further research is yet to be done to establish how other factors positively or negatively impact the use of PRP, double-blinded trials on outcomes with patients who are smokers versus non-smokers after PRP injections, and prospective studies on decreasing alcohol consumption, weight loss, and dietary changes would be of great benefit in assessing the interplay of PRP injections with other variables.

## Introduction

Knee osteoarthritis (kOA) and knee pain are increasingly prevalent pathologies presenting to primary care and secondary care alike, contributing to worsening financial burdens shouldered by the NHS. This retrospective study primarily aims to analyse the use of intra-articular platelet-rich plasma (PRP) injections in the treatment of knee pain, especially kOA, in a District General Hospital. Secondarily, the demographics of patients presenting with knee pain are assessed, and this paper puts forth a hypothesis as to why the population in the clinic is skewed away from an expected average. Finally, existing literature surrounding PRP injections for kOA and other knee pathologies is examined to discuss the rationale for their outcomes compared to this study.

Though this study is conducted in a secondary care setting, it’s crucial to recognise the significant impact it has on the burden placed on general practice (GP). In a study by Swain et al. [1], over 200,000 people with osteoarthritis (OA) were compared to an equal number of individuals without OA (the control group). The results showed that the OA group had a median of 10.91 primary care consultations per year following the index date, compared to 9.43 in the control group (p = 0.001). OA patients experienced higher rates of GP consultations, hospital admissions, and all-cause mortality, with variations across different joint sites. This highlights the need for a range of cost-effective strategies to manage OA and joint pain in order to alleviate the pressures on the healthcare system as a whole. PRP is theorised to contain a high percentage of various growth factors, which are hypothesised to induce vascularisation of the injected area [2] and stimulate healing of chondral tissue to allow symptomatic relief for kOA [3]. The burden of kOA is increasing. Between 1997 and 2017, there were 494,716 incident OA cases in patients aged over 20 years. The standardised incidence of any OA in 2017 was 6.8 per 1000 person-years (95% CI: 6.7 to 6.9), and prevalence was 10.7% (95% CI: 10.7-10.8%) [4]. The incidence of risk factors such as diabetes, obesity, and increasing average societal age is increasing; thereby, the risk of developing kOA in the population is magnified. The importance of developing cost-effective measures which are also minimally intrusive to patients to combat kOA and other common presentations of knee pain is critical to allow patients to return to their baseline level of independence and activity as efficiently as possible. The development of intra-articular injections for the knee allowed for a relatively easy and quick procedure in comparison to arthroplasties to give clinicians and patients options for non-invasive treatments. However, the outcomes need to be studied to assess whether patients can indeed be safely discharged from the clinic without being listed for total knee replacements (TKRs) or whether the adverse outcomes outweigh the benefits.

## Materials and methods

Study design and data collection

**Figure 1:**
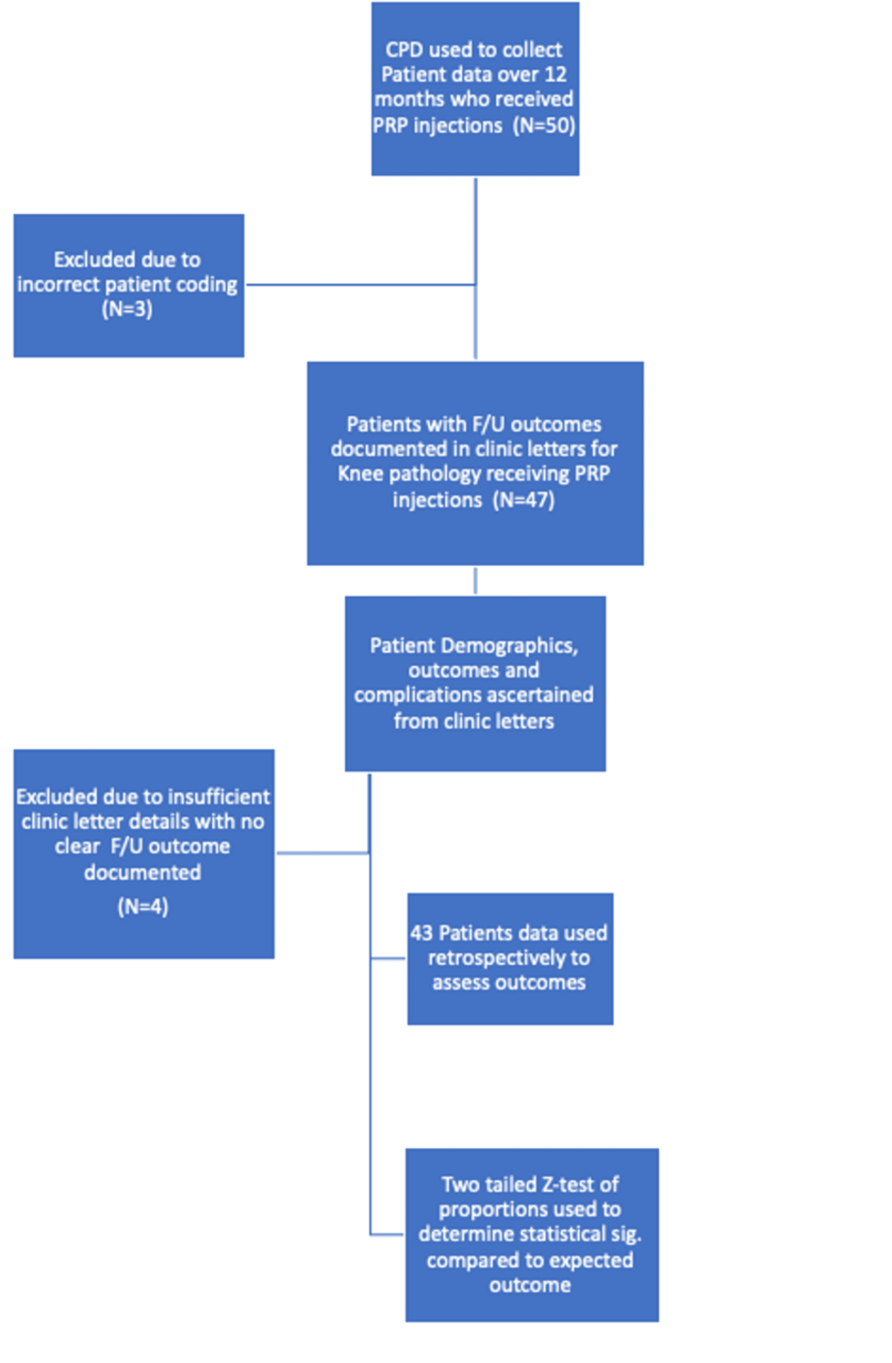
Study design flowchart F/U: follow-up; OA: osteoarthritis Three patients needed to be excluded as they were incorrectly coded as knee OA but were indeed injected for ankle pathologies, and four patients were excluded due to insufficient or unclear clinic letter details regarding the outcome

Using an elective clinic in a District General Hospital in the United Kingdom, we examined the outcomes between 2023 and 2024. Figure 1 shows the study design of the paper and the process of acquiring the patient cohort used in the analysis. We have retrospectively used follow-up outcomes to analyse the efficacy of the treatment. The patients included in the paper had Kellgren-Lawrence (K/L) grades 1 to 3 (mild to moderate) severity of OA.

We analysed whether these patients could be successfully discharged, defined as discharged without further recall or attendance; only needed a telephone follow-up; or had to be listed for further treatment (including arthroscopy, repeated intra-articular injections, imaging, or arthroplasty). Initially, 50 patients were included in the study; however, three needed to be excluded due to incorrect patient coding. Once the data were collected, four patients needed to be excluded due to insufficient details outlined in the clinic letters. Incorrect coding is defined as patients who were wrongly coded as knee pathologies, but upon further investigation had received PRP injections for ankle pathologies. 

Patients’ subjective analysis of their own experience, defined as improving, equivocal to prior pain, or worsening, information recorded in the clinic letters, is also discussed. Arguably, patients’ own symptoms offer the most insight into a treatment’s efficacy with regard to decreasing future costs to the NHS, as they would thereby be less likely to need further care in the primary or secondary care settings, regardless of plain, film, or histopathological evidence. 

A total of 43 patients (33 women; 10 men) were included in the study. The data was collected for patients presenting to the knee clinic in a single District General Hospital in the UK between 1 January 2023 and 1 January 2024 over a period of 12 months. The duration of relief, if any, was reported and was followed up to assess whether it had lasted <3 months, 3-6 months, 6-12 months, or over one year. Data was collected using clinic letters retrospectively using our Internal Electronic Patient Record System. 

Statistical analysis

MS Excel (Microsoft Corporation, Redmond, Washington, United States) was then used to store and analyse the data with the employment of a two-tailed Z-test of proportions to obtain a p-value and thereby determine statistical significance. The two-tailed Z-test was chosen with the null hypothesis being that there is no difference between the two population proportions, whereby p < 0.05 is the value to determine a statistically significant difference between the proportions of the populations being evaluated.

## Results

Analysing the demographics of the cohort, 37 out of 43 patients had the full three-course of injections (86.05%), three patients had two injections (6.98%), and three patients only needed one injection (6.98%). Table 1 displays the differences in sex distribution of the population included in the study. The number of women presenting at the clinic was significantly higher than men (33 women vs 10 men, p < .00001). Table 4 above demonstrates the duration of relief that the patients had gotten from the PRP injections. The duration is categorised into less than three months, 3-6 months, 6-12 months, or more than 12 months. A total of 41/43 patients (95.34%; p = 0.0308; p < 0.05) got some form of relief from the injections, which is a statistically significant proportion of the cohort. There are no significant differences in the durations of relief for the aforementioned time periods. Table 5 demonstrates the duration of relief that the patients had gotten from the PRP injections. The duration is categorised into none, less than three months, 3-6 months, 6-12 months, or more than 12 months. A total of 41/43 patients (95.34%; p = 0.0308; p < 0.05) got some form of relief from the injections, which is a statistically significant proportion of the cohort. There are no significant differences in the durations of relief for the cohort of patients who had more than 0 (i.e., some form of relief) for the aforementioned time periods <3 months vs 3-6 months vs 6-12 months vs >12 months; 34.88% vs 25.58% vs 18.60% vs 16.23%; p > 0.05). 

**Table 1:** Demographics of study cohort; female proportion found to be significantly higher than men (p < 0.00001)

Sex	Number of patients	Percentage (%)
Male	10	23.26
Female	33	76.74

The average age of the study group was 51.16 years (±3.912; 95% confidence intervals). The variance of the pathological presentations is listed in Table 2. The majority of the patients had presented as kOA, followed by patients with a history of soft tissue trauma, defined as meniscal or ligamentous injury without a fracture, chondromalacia patellae (CMP) patients, and idiopathic knee pain. Of the 23 patients who had presented with kOA, seven had grade 1 OA, nine had grade 2 OA, and seven had grade 3 OA. A total of 16/23 had very mild to mild OA, and seven had moderate OA. Out of the 23 patients who had presented with kOA, 10 (43.48%) were able to be discharged or just needed one telephone follow-up (TFU), nine (39.13%) needed further investigations or FTF, and four (17.39%) were listed for a TKR. 

**Table 2:** Diagnosis of patients presenting to the clinic; kOA proportion is significantly higher than the remaining diagnosis (p = 0.00672; p < 0.05) kOA: knee osteoarthritis

Diagnosis	Number of patients	Percentage (%)
kOA	23	53.49
Chondromalacia patellae (CMP)	6	13.95
Post soft tissue trauma	8	18.60
Idiopathic pain	6	13.95

The distribution of kOA patients with their respective grades and outcomes is displayed in Table 3. It is evident that patients presenting with mild OA (grades 1-2) were more likely than those with moderate to severe OA (grade 3) to have a successful discharge. Out of 16 patients with grades 1-2 OA, nine had a successful outcome (56.25%) compared to 1/7 patients with grade 3 OA (14.29%); this is a statistically significant difference in outcome (9 vs 1; 56.25% vs 14.29%; p < 0.05). 

**Table 3:** The Kellgren-Lawrence (K/L) grade distribution and outcome for each grade FTF FU: face-to-face follow-up; PRP: platelet-rich plasma; TKR: total knee replacement A successful outcome is defined as being discharged from the clinic with no further follow-up or only needing a TFU

Knee osteoarthritis severity (K/L score)	Number (%)	Successful outcome N (%)	Listed for TKR N (%)	FTF FU/ PRP N (%)
1	7 (30.43)	3 (42.86)	0 (0)	4 (57.14)
2	9 (39.13)	6 (66.67)	1 (11.11)	2 (22.22)
3	7 (30.43)	1 (14.29)	3 (42.86)	3 (42.86)

Table 4 below highlights the outcomes for the patients. A successful outcome in this study is defined as being discharged successfully or only needing a TFU and subsequent discharge. A total of 20 patients (20/43; 46.51%) had successful outcomes. This is a statistically significant proportion of the cohort (p = 0.0423; p < 0.05). A total of 19 patients (19/43; 37.21% needed further investigations or an FTF. The FTF appointments were made due to a lack of improvement with the combination of conservative measures such as simple physiotherapy, simple analgesia, and PRP. These patients needed FTF to be re-assessed, and 4/19 were listed for MRI scans, which showed soft tissue injuries, needing consideration of arthroscopic treatment. The remainder of the patients needing FTF were reconsidered for other differentials and needed hip and ankle assessments as possible causes of referred knee pain due to lack of improvement despite treatment. Three patients (3/43; 6.98%) needed further PRP injections beyond the initial planned three doses. Four (4/43; 9.30%) patients were listed for a TKR. As shown in Table 3, after the respective course of injections, 14 patients (32.56%) were successfully discharged from the clinic, and a further six only needed TFU (13.95%). A total of 16 patients (37.21%) needed a further FTF or investigations, and three patients (6.98%) needed further PRP injections. The number of patients needing further PRP injections was significantly less than the proportion of patients with a successful outcome (3 vs 20; 6.98% vs 45.51%; p < 0.05). 

**Table 4:** Proportion of patients who had variable outcomes from the clinics. The number of patients discharged or only needing a TFU was significantly less (p < 0.05) than patients needing further investigations FTF: face-to-face; FU: follow-up; PRP: platelet-rich plasma; TKR: total knee replacement

Outcome	Number of patients	Percentage (%)	p- value
Discharged or Tel FU	20	46.51	0.0423
FTF FU/ further investigations	19	37.21	0.23014.
listed for PRP injections	3	6.98	0.0226.
listed for TKR	4	9.30	0.0536

Table 5 below demonstrates the duration of relief that the patients had gotten from the PRP injections. The duration is categorised into none, less than three months, three to six months, six to 12 months, or more than 12 months. A total of 41/43 patients (95.34%; p = 0.0308; p < 0.05) got some form of relief from the injections, which is a statistically significant proportion of the cohort. There are no significant differences in the durations of relief for the cohort of patients who had more than 0 (i.e., some form of relief) for the aforementioned time periods <3 months vs 3-6 months vs 6-12 months vs >12 months; 34.88% vs 25.58% vs 18.60% vs 16.23%; p > 0.05). 

**Table 5:** The duration of relief defined as 0 (none), less than three months (<3 months), between three and six months (3-6 months), six to 12 months (6-12 months), or over one year (>12 months)

Duration of relief	Number of patients	Percentage (%)	p-value
0	2	4.65	0.0308
<3 months	15	34.88	0.1868
3-6 months	11	25.58	0.5352
6-12 months	8	18.60	0.8728
>12 months	7	16.23	0.6527

## Discussion

Suggesting mechanisms of action 

PRP is harvested from a patient by taking blood from the patient via venepuncture within a sterile environment. This sample is placed in a centrifuge machine to separate the erythrocytes from the platelets. The location for injection is prepped and draped as necessary. The PRP obtained is re-injected into the joint space, either with a superolateral or inferolateral approach to the knee joint under local anaesthetic. The patient is usually discharged on the same day as an elective day-case procedure. Repeat injections are usually listed for 4-6 weeks. PRP is theorised to contain a high percentage of various growth factors, such as fibroblast growth factor, epidermal growth factor, vascular endothelial growth factor, transforming growth factor-β, and platelet-derived growth factor [2]. 

Examining the constituents and mechanisms of PRP action in more depth, Wu et al. [3] looked at the in vivo action of PRP in rabbits. In order to investigate whether PRP has a positive effect on chondrogenesis in vivo, Wu and colleagues generated PRP/chondrocyte composites, which were injected into the subcutaneous tissue of the donor rabbits. After two months, tissues were formed under the skin, as MRI evaluation showed the presence of cartilage-like tissue. These findings suggested that PRP could be used as a cell scaffold in cartilage tissue engineering.

The burden of kOA

It is prudent to employ cost-effective measures to treat patients with accessible and relatively non-invasive methods to achieve similar outcomes as more complex methods, such as arthroplasty or soft tissue reconstruction. The incidence of kOA is expected to increase, given factors such as increasing obesity rates and physical inactivity, which contribute significantly to musculoskeletal conditions. Approximately one in five adults (18.2%) over 45 years of age in England has kOA [5]. The effective treatment of knee pain extends beyond the fiscal efficiency of our healthcare system to increasing the quality of life years for patients. Dakin et al. [5] studied 34 hospitals within the United Kingdom to assess the quality-adjusted life years (QALYs) observed in TKR. They found, on average, primary TKR and five years of subsequent care cost £7,458 per patient, and patients gained an average of 1.33 (SD: 1.43) QALYs. TKR is highly cost-effective for most current patients if the NHS is willing to pay £ 20,000-£30,000/QALY gained. 

How PRP injections may relieve some of this burden

The rationale for using PRP injections is twofold: it offers patients an alternative to arthroplasty, which comes with a plethora of potential adverse outcomes and complications; and it offers a delayed need for arthroplasty to maximise the years of functional mobility it can provide a patient. In our study, it is evident that PRP was effective for patients presenting with mild severity of OA rather than moderate to severe grades. While this may seem limited, indeed, these were patients who had already trialled conservative measures and analgesia. If the majority of patients with mild OA could be effectively treated with the addition of PRP to more conservative measures, and this allows for a greater discharge rate and decreased burden in secondary and indeed primary care settings, then this is of great benefit. Out of 16 patients with grades 1-2 OA, nine had a successful outcome (56.25%) compared to 1/7 patients with grade 3 OA (14.29%); this is a statistically significant difference in outcome (9 vs 1; 56.25% vs 14.29%; p < 0.05). While TKRs offer a largely positive experience for patients, allowing for the majority to regain lost functionality, complications can unfortunately occur. Nham et al., who conducted a retrospective study of 5,901,057 TKR, found that infection (24.62%) was the most frequent reason for revision surgery, followed by mechanical complications (18.62%) and dislocation (7.67%) [6].

Looking at the existing literature regarding PRP injections, there is some encouraging evidence of PRP efficacy, which corroborates this paper’s findings. Though this is to be approached with caution, as outcomes can be dependent on a variety of factors other than the PRP injection itself, the existing studies are encouraging. A retrospective study in the United States looking at the time to a TKR post-intra-articular injection compared PRP to corticosteroids (CS) and hyaluronic acid (HA) [7]. The PRP cohort had a total population of 3,240 patients, of which only 2.2% received a subsequent TKA compared to the corticosteroid cohort, of whom 5.9% received a subsequent TKA [7].

A systematic review was performed to evaluate maximum medical improvement in patients undergoing injections of different modalities for kOA. Demographic factors of the patients being reviewed were analysed, with patient-reported outcomes as reported by the visual analog scale (VAS). Overall, 79 studies were utilised with 8,761 patients. CS injections and PRP injections reached their maximum pain control at four to six weeks after injection [8]. This finding was mirrored by two of the patients from our cohort who were initially listed for a course of three injections for idiopathic knee pain. They had relief prior to the second injection and had been successfully discharged from the clinic and not re-presented due to the high efficacy of the PRP injection in their particular cases. PRP in their study demonstrated the most prolonged pain relief relative to the other injection types. 

PRP for CMP and other knee pain causes

Table 2 highlights the importance of recognising other causes of presentation to the knee clinic other than OA. In this study, there were six patients (6/43; 13.95%) who were treated with PRP injections for CMP. CMP is used to describe the softening of patellar articular cartilage and is a broadly defined pathological mechanism. Even though there were more patients presenting with kOA who could be discharged or needed only TFU, this was not statistically significant (p = 0.48392). However, patients who were treated for CMP did have a significant difference (p = 0.0042) in those who had PRP and could be discharged/TFU versus those who needed further investigations/treatment or were listed for TKR. No patients who presented with idiopathic knee pain or CMP had to be listed for TKR.

Ostojic et al. aimed to investigate the effect of injectable PRP on patients with anterior knee pain in the absence of altered patellofemoral joint anatomy [9]. 43 patients of the affected population were recruited to participate in this non-randomised controlled trial, 28 patients in the injection group and 15 in the only physiotherapy group. While patients in the experimental group received three PRP injections and one injection of HA, comparators underwent only physiotherapy as per the established local protocol. Outcomes were measured at baseline, and after three and six months of the treatment. They found a statistically significant difference favouring the injection of PRP over the physiotherapy-only group was observed (p < 0.001). The superiority of the therapeutic modality under investigation was observed at three and six months after the initial diagnosis was made. Furthermore, the results of this study revealed a significant improvement at three and six months when compared to baseline measures. Interestingly, they found that younger patients benefited more than the older population when compared after negating the remaining variables. 

From our study, we had 75% (6/8; p = 0.00424) patients who had PRP injections as a way to treat CMP who were discharged from our clinic, which highlights potential further use of PRP in treating other causes of knee pain other than kOA. Furthering this, it would be prudent to look at the literature concerning PRP treatment of soft tissue injuries of the knee. 

Örsçelik et al. also examined the use of PRP for treating CMP [10]. Prolotherapy (PrT) is a regenerative injection technique used for chronic musculoskeletal disorders. The study aimed to compare the effectiveness of PRP and PrT in treating CMP. Seventy-five patients with CMP symptoms, who had not responded to three months of conservative treatment, were included in the study. Pain scores were measured at the time of injection and reassessed at three and six weeks, as well as 12 months after treatment. All participants were prescribed a standard 12-week exercise program. The results showed that both PRP and PrT significantly improved pain and knee function after a minimum of one year of follow-up (p < 0.05). However, PRP was found to be more effective than PrT, particularly in reducing pain during exercise, improving range of motion, decreasing crepitus, and reducing the total number of analgesics needed by the patient.

In the early stages of tendon healing, cytokines like transforming growth factor-beta (TGF-β) play a key role in the formation of new blood vessels and collagen synthesis. These growth factors are found in high concentrations in PRP and may be particularly beneficial during the initial stages of tendon repair.

A prospective study [11] investigated the effectiveness of PRP injections in patients with chronic patellar tendinopathy. A total of 15 patients were treated with multiple PRP injections and physiotherapy, compared to 16 patients who received only physiotherapy. The group receiving PRP showed a statistically significant improvement in their activity levels, measured six months after treatment. Additionally, those treated with PRP reported a significant reduction in pain during daily activities, work, and sports.

In this paper, three patients had known meniscal injuries that were initially treated with arthroscopic meniscal repair, two of whom were successfully discharged after receiving PRP injections for pain management. The meniscus, primarily composed of type 1 collagen, plays a crucial role in shock absorption, proprioception, and joint stability. The outer part of the meniscus is vascular, offering better potential for healing than the inner, avascular zone. Early studies suggest that PRP may aid in healing by stimulating cell proliferation and vascularisation, particularly in the meniscus. For healing to occur, a blood supply is necessary, which is absent in the inner white-white zone [11]. Therefore, the vascularisation induced by PRP could be essential for promoting meniscal repair.

Moretti et al. [12] conducted a prospective study not only to assess the efficacy of PRP injections in patients with kOA but also to assess radiological outcomes. A total of 153 patients received three consecutive PRP injections and completed follow-up evaluations. The study evaluated three different knee pain scores before the PRP injection, as well as one, three, and six months after treatment. Additionally, all patients underwent MRI and X-ray assessments both at baseline and after six months. The results showed a statistically significant reduction in pain scores (p < 0.05). However, MRI scans did not reveal statistically significant improvements in cartilage thickness for either the tibial plateau or femoral cartilage. No radiographic changes were observed in any of the patients. While radiological assessments did not show mechanical improvements, the study concluded that PRP injections are a valid conservative treatment option for reducing pain and improving the subjective quality of life for patients, with benefits evident at the six-month follow-up.

Does course length affect outcome?

Interestingly, all of the patients in this study were due to receive a course of three injections as per the initial clinic letter outlining the plan for their treatment. Of the 43 patients, 37 had the full three-course injections (86.05%), three patients had two injections (6.98%), and three patients only needed one injection (6.98%). Only 86% received the full course. Out of the remaining six patients, four had self-discharged due to the pain improving drastically, one was unable to have the third due to venepuncture difficulties, and the remaining patient had responded poorly to the initial PRP injections and was subsequently listed for TKR. This paper did not find any association between the number of injections used with the outcome; however, the number of patients receiving one or two injections rather than three was too small to draw valid comparisons. 

There may be a difference in response depending on the number of injections used. It could be hypothesised that more injections do not have increased efficacy, rather that the pain improvement is a curve graph rather than a linear relationship to the number of PRP injections. To explore this hypothesis further, we can look at the surrounding literature. Zhuang et al. [13] looked at 120 patients with comparable severities of kOA who were randomly assigned to three groups: 106 patients who received a single injection of PRP; three injections or five, all spaced one week apart. The patients’ conditions were evaluated using pain scores at baseline and at intervals up to one-year follow-up. 

They found that employing three and five injections of PRP was substantially more effective than a single injection in reducing knee pain and stiffness, as well as enhancing physical function in patients with kOA. Importantly, they found that no statistically significant difference was observed between three and five injections at all follow-up intervals.

Görmeli et al. [14] studied 162 patients with kOA, who were randomly assigned to different treatment groups. These groups received either three doses of PRP, a single dose of PRP, one dose of HA, or a saline injection (control). The patients were assessed before the injections and again at the six-month follow-up. The results revealed a statistically significant improvement in pain scores across all treatment groups compared to the control group. Notably, patients who received three PRP injections showed significantly better knee scores than those in the other treatment groups. Interestingly, the severity of kOA influenced the response to the intra-articular injections. In the early-stage OA subgroup, patients who received three PRP injections had significantly better clinical outcomes, while no significant differences were found in clinical results among patients with advanced OA across the treatment groups.

Sex differences in regards to kOA and knee pain

In our cohort, Table 1 shows the proportion of women found to be significantly higher than men (33 women vs 10 men, p < 0.00001) who had been referred to the knee clinic for OA. The cause is multifactorial and includes anatomic differences, involving the mechanical and anatomical axis differences of the joints above (hip) and below (ankle). The anatomic differences within the knee joint between males and females that may play a role include narrower femurs, thinner patellae, larger quadriceps angles, and differences in tibial condylar size. This is reflected in the fact that TKR prostheses are differentiated on a sex basis. 

Hame et al. [15] also suggest that differences in knee cartilage volume are an important factor. Using analysis of magnetic resonance imaging (MRI), Hanna et al. [16] observed that men had significantly greater total tibial and patella cartilage volume when compared to women. Women also had a higher prevalence of patellar cartilage defects at baseline, which are contributing factors to the increased presence of kOA in women.

The excess risk for OA in women is also supported by a systematic review and meta-analysis that revealed the prevalence of kOA is higher in women (21.7% (95% CI: 19.0%-24.5%)) than in men (11.9% (95% CI: 10.2%−13.8%)) with a greater difference between sexes for each decade starting at age 40 [17]. The decline in oestrogen levels after menopause has been implicated as a potential factor in the increased risk for OA in women. Oestrogen has been shown to protect against cartilage degradation. Post-menopause, oestrogen levels decline significantly, which could potentially elevate risk for incident OA. 

There are numerous anatomic and biomechanical factors that differ between women and men, including limb alignment (e.g. wider pelvis and greater quadriceps angle (Q angle-the angle of pull on the patella). Perhaps, due in part to the wider pelvis and more extreme Q angle, women have greater malalignment and varus-valgus laxity than men, leading to different stressing loads placed across the knee joint over time, leading to different patterns and severity of kOA compared to men.

TFU versus FTF

Cost-effectiveness is a complex issue and challenging to evaluate in a single-centre trial. Existing literature highlights potential cost-savings that are theoretically robust and applicable across various medical specialties. Akobeng et al. [18] conducted a randomised controlled trial involving 86 paediatric patients with inflammatory bowel disease at Manchester Hospital (UK). Their study found that telephone consultations averaged £35.41 per patient, compared to £51.12 for FTF consultations, a cost difference of £15.71 per consultation. Fink et al. [8] conducted a separate randomised trial with 123 general surgery outpatients, finding that telemedicine consultations were significantly shorter than FTF visits (telemedicine: 10.52 ± 7.2 minutes; FTF: 15.95 ± 9.96 minutes; p = 0.0021) while still achieving acceptable levels of patient satisfaction.

Cautious moving forward

It is shown in this study that the majority of patients benefit somewhat from PRP injections. Table 5 shows that 41/43 patients (95.34%; p = 0.0308; p < 0.05) got some form of relief from the injections, which is a statistically significant proportion of the cohort. However, it is important to acknowledge that while some individual studies suggest PRP could be a promising solution for a complex pathology, there is evidence indicating that its effectiveness may not be as high as initially hoped. High-quality studies like the RESTORE trial have shown that PRP injections don’t work better than a placebo (saline) for reducing knee pain or preventing cartilage damage in people with kOA after one year. These results suggest that PRP’s effects might be no better than a placebo in some cases. Placebo-controlled trials are important to really understand how well treatments work. The RESTORE trial, led by Bennell et al. [19], provides strong evidence on this. In this study, 288 people with mild-to-moderate kOA received either three PRP injections or placebo injections over three weeks, with follow-up over 12 months. The results showed no significant difference between the PRP and placebo groups for the main outcomes. After a year, the pain level dropped by -2.1 points in the PRP group and -1.8 points in the placebo group, a difference that wasn’t statistically significant. Both groups showed similar improvements in function, and MRI scans showed no major difference in cartilage loss between the two groups. Both groups experienced slight cartilage loss over the year. This well-conducted study suggests that the benefits seen in earlier studies might have been due to the placebo effect or other factors unrelated to the actual PRP mechanisms of action. The authors pointed out that clinical guidelines didn’t recommend PRP at the time because there wasn’t enough evidence, and their findings supported a cautious approach.

Complications associated with PRP

In this paper, it is highlighted that any introduction of drugs into a joint space and the needle alone poses a risk of complications arising, either immediately intra-procedurally or in the follow-up period. We did not have any patients with complications arising from their PRP injections. Potential worries could have been the risk of infection, bleeding, worsening of the inflammation and pain, cartilage damage, and iatrogenic injury to the soft tissue or neurovascular bundle, in addition to the risks of venepuncture itself. This paper did not show any patients who had immediate complications, defined as pathologies caused by the procedure itself. Rather, they either did not respond to the treatment itself or, in one case, were unable to undergo the treatment due to vascular access issues. 

Arita et al. [20] conducted a literature review using PubMed to identify research articles related to adverse events associated with PRP therapy. The survey revealed that PRP therapy may be linked to several complications, including postoperative infections, inflammation, allergic reactions, and the development of nodules. The most frequently reported was, somewhat unsurprisingly, postoperative infection. Given that PRP therapy involves blood collection, processing, and administration to patients, it is suspected that microbial contamination may occur at some point during these processes, leading to bacterial infections. Additionally, because PRP cannot be sterilised, it is crucial to prevent microbial contamination throughout the treatment process. To better prevent microbial contamination, further investigation into the risk factors during the PRP treatment process is needed. Currently, most reports of AEs related to PRP therapy are case studies; thus, accumulating high-quality evidence and conducting thorough evaluations are essential to determine the causal relationship between PRP therapy and these adverse events.

Poor documentation of pain in the clinic letters

It is highlighted repeatedly throughout our journeys in medicine that documentation and clear communication are key, as they allow clarity in recollection of events, details, and assessments. In our paper, it was noted that a low number of clinic letters had pain scores on follow-up documentation. Rather, subjective scores from patients were taken. While, as explained earlier in the paper, these are invaluable, having objective measures allows monitoring of developing trends in the patients’ journey to recovery. This study emphasises an opportunity for improvement in clinicians' documentation of pain metrics. Ardon et al. [21] explored the effectiveness of combining an educational program with electronic prompting to encourage accurate documentation of pain scores. The study involved 98 patients, divided equally into a study group (49 patients) and a control group (49 patients). The study group included patients who underwent knee and hip arthroplasties after receiving an educational program on analgesia, which incorporated electronic prompts to remind clinicians to document pain scores and prescribe analgesia appropriately. The study specifically examined the frequency of documenting pain scores and preoperative opioid use. After implementing the educational initiative, 67% of patients in the study group had their baseline pain scores recorded in the preoperative acute pain service (APS) documentation, compared to just 20% in the control group (p = 0.0001). Additionally, preoperative opioid use was recorded in 24% of the control group’s documentation, but this figure rose to 73% following the educational intervention (p = 0.0001).

Limitations of the study 

During the design of the study, it became apparent that there may be some limitations. One limitation of the study is the relatively small sample size of 43 patients, which may result in anomalous results skewing the data. The study, therefore, would need to be repeated with a larger study population to better represent a more valid average. It would also be advisable for future studies to hold a control group, who did not receive any PRP injections, rather managed with simple analgesics alone, with the same grade of kOA, to draw comparisons across outcomes in regard to FU or duration of relief. The duration of the paper can be increased with the utilisation of a larger sample if the study were employed across multiple clinical sites across the country to assess how different methods of PRP delivery may impact the outcome. It is somewhat difficult to nullify the placebo effects on trials involving joint pain, given their subjective nature and how patients respond to treatments linked with their perceived efficacy. A future trial, if approved, could involve a double-blind study utilising a cohort injected with PRP, and another cohort with an unreactive, inert treatment not known to cause any adverse intra-articular effects, and study the long-term outcomes of these two groups.

## Conclusions

We have looked at the use of PRP in one hospital setting, while it is evident that PRP injections have their place in treating knee pain, with patients with mild grades of OA benefiting more than those with increased severity. PRP could potentially be beneficial for patients with chondral pathologies. Much further research is still needed for efficient ways of treating an ailment that affects millions of people worldwide. The limitations of the study above highlight that much work is yet to be done to study the true potential and long-term outcomes for PRP intra-articular injections.
